# Numerical Simulation of Rotational Speed Sinusoidal Pulsation for Enhancing Polymer Processing Based on Smoothed Particle Hydrodynamics

**DOI:** 10.3390/polym17030415

**Published:** 2025-02-04

**Authors:** Tianlei Liu, Hesheng Liu, Tianwen Dong, Jiamei Lai, Wei Yu, Zhong Yu, Huiwen Yu

**Affiliations:** 1 Jiangxi Key Laboratory of High-Performance Precision Molding, Polymer Processing Research Laboratory, Nanchang University, Nanchang 330031, China; liusya0618@163.com (T.L.); laijm@163.com (J.L.); 352400240017@email.ncu.edu.cn (W.Y.); 2 School of Mechatronics and Vehicle Engineering, East China Jiao Tong University, Nanchang 330013, China; 3 Jiangxi Province Key Laboratory of Applied Optical Technology, Shangrao Normal University, Shangrao 334001, China; 306150@sru.edu.cn; 4 School of Mechanical and Automation Engineering, Wuyi University, Jiangmen 529020, China; ddlianyi@163.com

**Keywords:** difference pulsating, particle distribution, variable field, smoothed particle hydrodynamics

## Abstract

Vibration-assisted methods are playing a more and more important role in processing polymers for twin screw extruders (TSEs) in order to satisfy the increasing requirements for polymetric products in various applications, but existing vibrating technologies are usually restricted in school laboratories and industrial research rooms because of their drawbacks. The purpose of this study is to design a novel vibration method for TSEs. Numerical simulation was performed based on a meshless method, namely smoothed particle hydrodynamics (SPH). The velocity distribution, particle distribution, and pressure of particles in a co-rotating twin screw component in the conveying zone of a TSE are investigated in detail to recover the influence of the rotational speed excitation on the flow properties of both fully filled and partially filled states. The results show that cases under superimposed excitation can produce a more variable physical effect, thus enhancing and weakening the velocity field and the pressure field alternately. And on the whole, that effect could improve the particle distribution in according cases. These findings can lay a solid foundation for further study on the development and application of superimposed excitation technology in the polymer processing of TSEs.

## 1. Introduction

The increasingly high requirements for polymeric products in various applications have been challenging for the processing and molding technologies used in present polymer industries. To solve product defects such as weld lines, sink marks, nonuniform internal stress, and so on, numerous investigations have been conducted by theoretical, experimental, and numerical methods [1,2].

Among these methods, the method of applying a vibration field is proven to be effective and efficient in bettering the plasticizing or molding process and improving the properties of polymeric products [3,4,5]. For example, experimental results [6] on an electromagnetic pressure vibration injection molding machine show that the filling time can be shortened from 2.15 to 1.95 s, and the crystallinity can increase from 28.5% to 30.1% by changing the frequency. And experiments [7] using ultrasonic vibration from an ultrasonic vibration extruder reveal that thermal effects and mechanical effects decrease the apparent viscosity of the polystyrene melt, accounting for 56% and 44%, respectively. Moreover, vibration-assisted injection molding by a controlled oscillation shear flow can reduce the cycle time by 40%, and increase the overall crystallization from 42% to 52–60% [8].

The most widely used excitation sources of vibration-assisted methods are mechanical, ultrasonic, and electromagnetic. And these excitation devices are usually superimposed onto the screw, die, or mold in parallel, perpendicular, and/or in the rotational direction relative to the melt flow [9,10,11,12]. However, these excitation sources are usually tested in small processing devices in school and industrial laboratories due to the large inertia of large-sized extruders and injection molding machines, which greatly limits the application of vibration technologies [13,14,15]. Different from the above vibration-producing methods, Liu et al. [16] put forward a new concept of a differential pulsating excitation system which produces a vibration field not by loading external vibration force on the polymer machines but by changing the rotational speed of the screw in a sinusoidal way. Statistical results [17] by numerical simulation on a simplified screw element indicate that the introduction of a speed pulsating field under certain parameter settings can enhance the stretching rate, reduce the separation scale, increase the mixing efficiency, and lower the screw drive force.

Moreover, this method makes it possible to apply a vibration field to a twin screw extruder (TSE). Even though there are many studies on TSEs conducted mostly by numerical simulation with computational fluid dynamics (CFD) methods, such as the finite difference method (FDM), finite volume method (FVM), and finite element method (FEM) [18,19,20,21,22], reports on vibration application to TSEs have not been seen in any printings. On the one hand, the complex geometry and fluids make it hard to conduct experiments with the vibration device on TSEs, which leads to a lack of researchers studying the problem with numerical simulation. On the other hand, although the CFD method has been employed in numerous aspects such as stitched composite laminates [23], twin screw extruder mixing [24], electrical machine cooling [25], and 3D magnetic field analysis [26], many drawbacks appear in simulating free surface flow and multi-phase flow, especially for tracking the interface between phases [27,28,29,30]. The method provided by LIU et al. does not depend on the geometry, which makes it suitable for either simple or complex models. Therefore, this method can be used to introduce vibration fields into experimental and simulation studies on the TSE.

For the second problem mentioned above, one effective solution is to use the smoothed particle hydrodynamics (SPH) method, which simulates flows using particles instead of meshes. The SPH method is perfect for overcoming the drawbacks of the mesh-based method, which is limited by problems associated with the extreme deformation caused by forced boundaries or small gaps between complex models. The excellent adaptive properties of SPH have increasingly attracted the attention of scientists and engineers from various research directions, such as astronomy, engineering mechanics, explosions, aviation, and naval engineering [22,31,32]. These research works in turn promote the SPH method and greatly expand its application. As for polymer processing by TSEs, recent studies [33,34,35,36,37] have mostly focused on continuous improvement in calculation efficiency, stability, and boundary treatment by performing experiments or simulations on free surface flows, single-phase flows, and multi-phase flows in fully filled or partially filled models.

In this paper, a rotational speed differential pulsating system on a TSE was proposed to provide vibration excitation, the algorithm for the excitation was implemented in the SPH open-source software DualSPHysics, and the influences of superimposed excitation on the flow properties of simplified, Newtonian, isothermal fluids in TSE were simulated by DualSPHysics. The main content of this paper can be outlined as follows: in Section 2, the models and method used in this study are introduced in detail, including the differential pulsating system, the geometric model, the governing equations, the parameter setting, the boundary conditions, and the computation information. Then, in Section 3, the results from numerical simulation work on fully filled and partially filled flows are contrastively discussed and analyzed between the states with and without rotational speed sinusoidal pulsation excitation. Finally, the obtained results from Section 3, as well as the limitations and future prospects of the current research work, are used to form conclusions in Section 4.

## 2. Methods and Implementation

### 2.1. Differential Pulsating System

**Figure 1:**
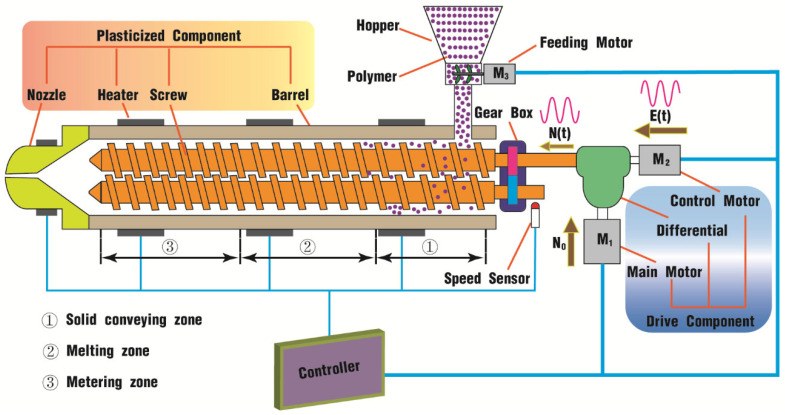
Schematic diagram of differential pulsation system in TSE.

As illustrated in Figure 1, the rotational screw differential drive pulsation system mainly consists of the plasticized component, the drive component, and the controller. The plasticized component includes a barrel, twin screws, a nozzle, and some heaters fixed along the surface of the barrel and the nozzle. Typically, the twin screws can be divided into three zones, that is, the solid conveying zone, the melting zone, and the metering zone. At the beginning of the solid conveying zone is a feed-well with a hopper, which is equipped with a feed motor (M3) for polymer materials so as to couple with the changing screw speed. The twin screws are coupled by a gear box, and the tail end of one screw is joined to the drive component by a differential.

The differential drive component contains a major motor (M1), a control motor (M2), and a differential. The motor M1 provides a basic speed *N*_0_ as the total input of the differential; the motor M2, which is connected to the right half shaft of the differential, serves as an input excitation, denoted as *E(t) = Asin (2πft)*, where *A* is the vibration amplitude and *f* is the vibration frequency. The left half shaft of the differential outputs the summation of the above two speeds to the screws of the TSE plasticizing system, recorded as *N(t)*. The screw speed can be detected with a speed sensor and thus can be controlled directly by adjusting the input speeds of motors M1 and M2.

Finally, the detection signals from motors M1, M2, and M3, the heaters, and the speed sensor are transmitted to the control center. Then, the controller analyzes the signals and sends instructions back to maintain the twin screws to run in a sinusoidal pulsation way and to keep the whole system effective and efficient.

Different from the literature [17], in which the unit of screw speed is revolutions per minute (RPM), the unit for rotational speed in DualSPHysics is in degrees. Thus, the unit of the excitation *E(t)* is degrees per second, and the screw speed *N*_0_ is supposed to be converted from RPM to degrees per second, that is, 360°/60 *N*_0_ = *6N*_0_ degrees per second.

The limitation for the parameters of the excitation can be given as follows (see details in Appendix A):

(1) $$Af\leq 3N_{0}/\pi$$

where *A* is the vibration amplitude and *f* is the vibration frequency for the vibration excitation; *N*_0_ is the basic rotational speed of the twin screws.

### 2.2. Geometric Model

**Figure 2:**
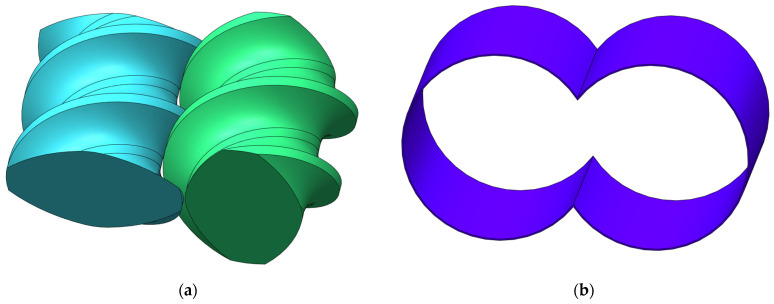

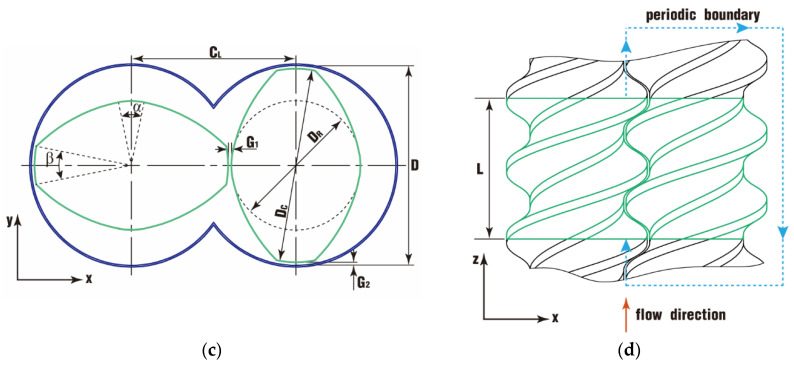
Configuration of simulation models: (**a**) twin screws; (**b**) barrel; (**c**) front view of the twin screw component; (**d**) top view of the twin screws.

As introduced in Section 2.1, the twin screws are typically split into three zones: a conveying zone, a melting zone, and a metering zone. The geometric model used in this study is located in the conveying zone, as shown in Figure 2. Figure 2a depicts a pair of double-headed and right-handed screw elements for a co-rotating twin screw extruder, and the twin screw marked in cyan is horizontally placed while the one in green is vertically placed. All the geometric models are generated in the STL (Standard Triangle Language) format by the commonly used software SolidWorks. Both of the twin screws consist of dynamic boundaries which provide movement power for the polymer particles flowing through the channel. Figure 2b describes a barrel matching the twin screws, and the barrel is the fixed boundary which forms the channel together with the twin screws.

The front and top views of the twin screw component are illustrated in Figure 2c and Figure 2d, respectively. Figure 2c gives the detailed schemes for the model, and Figure 2d describes the flow direction together with the periodic boundary setting. Here, one lead length of the twin screw component is selected for the simulation models (marked in green in Figure 2d). The algorithm implementation of one periodic boundary treatment in DualSPHysics brings much convenience for the simulation because this method makes it possible to simulate only one lead of twin screws with the same effect as simulating the whole screws, which is rather time- and memory-consuming.

Table 1 shows the geometrical parameters for the twin screw component dimensioned in Figure 2c,d. In addition, in order to be closer to real industry processes, a clearance of 1 mm is set between the twin screws and the barrel, denoted as *G*_1_ and *G*_2_, respectively.

### 2.3. Governing Equations and SPH Algorithm

The Navier–Stokes equations written in Lagrangian form are given by [22,38]

(2) $$\frac{d\rho }{dt}=-\rho \nabla \cdot v$$

(3) $$\frac{dv}{dt}=-\frac{1}{\rho }\nabla p+\frac{1}{\rho }\nabla \cdot \tau +F$$

where ρ, ***v***, p, τ, and F are the density, velocity vector, pressure, shear tensor, and body force, respectively.

In the SPH method, the entire system is represented by a finite number of particles that carry individual mass and occupy individual space [38]. This is achieved by particle approximation, and the particle approximation for a function and its spatial derivative can be written as

(4) $$f(x_{i})=\sum_{j=1}^{N}\frac{m_{j}}{\rho_{j}}f(x_{j})\cdot W_{ij}$$

(5) $$\nabla \cdot f(x_{i})=-\sum_{j=1}^{N}\frac{m_{j}}{\rho_{j}}f(x_{j})\cdot \nabla W_{ij}$$

where

(6) $$W_{ij}=W(x_{i}-x_{j})$$

(7) $$\nabla_{i}W_{ij}=\frac{x_{i}-x_{j}}{r_{i}-r_{j}}\frac{\partial W_{ij}}{\partial r_{ij}}$$

Here, *N* is the total number of particles in the support domain of particle *i*, $m_{j}$ and $\rho_{j}$ are the mass and density of particle *j*, $W_{ij}$ is the smoothing function of particle i evaluated at particle *j*, h is the smoothing length, vector $r_{i}$ and $r_{j}$ are the positions of particles *i* and *j*, and $r_{ij}$ is the distance between particles *i* and *j*.

The smoothing function $W_{ij}$ in Equation (6) is selected in the Wendland form with compact support of 1.2*h* in this work:

(8) $$W(R,\;h)=\alpha_{d}\times \left\{\begin{array}{l} (1-\frac{R}{2}{)}^{4}\times (2R+1),\;\;\;\;\;0\leq R<1;\\ 0\;\;\;\;\;\;\;\;\;\;\;\;\;\;\;\;\;\;\;\;\;\;R\geq 2, \end{array}\right.\;\;\;$$

where $R=\left|r_{i}-r_{j}\right|/h$, and the normalization constant $\alpha_{d}$ is equal to $7/(4\pi h^{2})$ in 2D and *21*$/(16\pi h^{3})$ in 3D.

Generally, there are two types of SPH algorithms to simulate the fluid flow, the weakly compressible SPH (WCSPH) and the incompressible SPH (ISPH) [33]. WCSPH is an explicit algorithm that is easy to program, and is also an effective and widely used way to simulate incompressible flows in a weakly compressible method. To account for the WCSPH, the Delta-SPH is proposed by introducing a diffusive term to reduce the density fluctuations, and then based on Equations (4) and (5), discretized governing Equations (2) and (3) can be given as [33,39,40]

(9) $$\frac{d\rho_{i}}{dt}=\sum_{j=1}^{N}m_{j}\cdot v_{ij}\cdot \nabla_{i}W_{ij}+2\xi hc\sum_{j=1}^{N}\left(\rho_{i}-\rho_{j}\right)\frac{r_{ij}\cdot \nabla_{i}W_{ij}}{{r_{ij}}^{2}+0.01h^{2}}\frac{m_{j}}{\rho_{j}}$$

(10) $$\frac{dv_{i}}{dt}=-\sum_{j=1}^{N}m_{j}\left(\frac{p_{i}}{\rho_{i}^{2}}+\frac{p_{j}}{\rho_{j}^{2}}\right)\nabla_{i}W_{ij}+\sum_{j=1}^{N}\frac{m_{j}(\mu_{i}+\mu_{j})r_{ij}\cdot \nabla_{i}W_{ij}}{\rho_{i}\rho_{j}({\left|r_{ij}\right|}^{2}+0.01h^{2})}v_{ij}+F$$

where the Delta-SPH diffusive coefficient *ξ* = 0.1 is used, *c* is the sound speed, $\rho_{i}$ is the density of particle *i*, $v_{i}$ is the velocity of particle *i*, $v_{ij}=v_{i}-v_{j}$, $r_{ij}=r_{i}-r_{j}$, μ is the dynamic viscosity coefficient, and $\mu_{i}$ and $\mu_{i}$ are the dynamic viscosity coefficients of particle *i* and particle *j*, respectively.

In a weak-compressibility situation, the pressure for particles is calculated directly from the density based on an equation of state [22,34]:

(11) $$p=B\left[{\left(\frac{\rho }{\rho_{0}}\right)}^{\beta }-1\right]+p_{0}$$

where $\rho_{0}$ and $p_{0}$ are the reference density and background pressure, respectively. β is a constant, usually β = 7; *B* is the pressure scale factor, usually set to $c^{2}\rho_{0}/\beta$, where *c* is the sound speed.

In the above discrete SPH equations, time integration is performed by a two-state symplectic method, and the timestep *dt* is controlled by three stability constraints: the Courant–Friedrichs–Levy condition, mass force term, and viscous diffusion term [41,42,43].

(12) $$dt=\min\;\left(0.25\frac{h_{i}}{c},\;0.25\min{\left(\frac{h_{i}}{f_{i}}\right)}^{1/2},\;0.125\frac{h^{2}}{\nu }\right)$$

where $h_{i}$ and $f_{i}$ are the smoothing length and acceleration of particle *i*, respectively; ν is the kinematic viscosity.

Based on the experiment on a co-rotating twin-cam mixer [44], we simulated the flow in the mixer by SPH and compared the results between the experiment and the SPH simulation [22]. The viscosity of fluid used in SPH is initially set to 10^−4^ of that in the experiment, which can speed up the calculation and not affect the simulation effect [22,45]. This setting also has other supporting evidence. According to the literature [36], the only limitation for the computational expense is the Reynolds number, and a typical viscosity value in the order of 1000 Pa.s would decrease the Reynolds number and increase the computational expense. In addition, our study in the literature [37] shows that only one case with a viscosity of 1000 Pa.s and less than 8 hundred thousand particles could cost up to 360 h, so the calculation time would take several months if the simulated models need about 10 million particles and the fluids are endowed with a viscosity in the order of 1000 Pa.s.

### 2.4. Parameter Setting

As stated in the above sections, the meshless method SPH employs particles to describe geometric models. And some little gaps in the models are required to have at least four layers of particles in order to improve the calculation accuracy [46]. In this study, since the smallest gaps between the screws and barrel are set to be 1 mm, the distance between particles (denoted as *dp*) is set to be 0.25 mm. As a result of the dimensions designed in Figure 2 and Table 1, it needs almost 10 million particles to represent the models in the full-filled state; then, in the half-filled state, there are about 5 million particles because only half the cavity of the fluid channel needs to be filled.

Such numerous particles, however, will lead to an extremely time- and memory-consuming calculation even though the parallel algorithm and the graphics processing unit (GPU) card are used together to speed up the calculation [37]. Moreover, a high viscosity can sharply lengthen this calculation [22]. In addition, it is more conducive to mixing for a two-phase flow when the two phases have the same viscosity [30]. Therefore, the viscosity ratio between fluid 1 and fluid 2 in this study is set to be 1, and the viscosity of fluids is initially set to a very low value of 1 Pa.s. In fact, the actual polymer is indeed a viscoelastic flow. This study simplifies the problem for the convenience of simulation. The simulated fluid is assumed as a Newtonian fluid with low viscosity rather than a non-Newtonian fluid with high viscosity. In other words, this study did not consider the influence of factors such as temperature, viscoelasticity, shear heating, and viscous dissipation on the flow property of the fluid, which would make the calculation extremely complex and time-consuming [36]. Moreover, because the main content of this paper is to introduce a novel vibration method for the twin screw extruder and to study the influence of the rotational speed excitation on the flow properties of both fully filled and partially filled states by comparing the results in the normal case without a rotational speed sinusoidal pulsation excitation, the interfacial tension and surface tension produced between different phases are not taken into consideration, which will be further researched together with the above influence factors in our follow-up study when the according SPH algorithms are solved.

Based on some numerical experiments in our past research [22,33,37,39], a sound speed of 20 m/s is accepted to balance the viscous force, which can prevent particles from penetrating the solid boundary. The smooth length *h* for the kernel function is typically set to 1.2 times that of *dp*. By substituting *c* and *h* into Equation (12), the timestep *dt* can be calculated: *dt* = 3.75 × 10^−6^ s.

As for the excitation field, the main content of this study is analyzing the effect of a vibration field on the flow property of the twin screw component compared with the normal condition without excitation, so only one pair of vibration parameters is adopted. The frequency *f* is set to be 5 Hz, and the amplitude *A* is set to 6 degrees accordingly, which complies with Equation (1).

To sum up, some key analysis parameter settings can be obtained from the above discussion, as shown in Table 2.

### 2.5. Boundary Conditions

There are two types of bodies used in the models drawn in Figure 2, and each body is composed of a group of SPH particles. One type is the solid bodies, composed of the barrel, which is stationary, and the twin screws, which are moving. The other type is the fluid body, that is, the space filling between the barrel and the screws.

Dynamic boundary conditions are loaded to handle the complex surfaces of the twin screw component [22]. A no-slip boundary condition is used for fluid particles near the surfaces of the barrel and the twin screws. The rotation of the screws is the power source of all the movements for the above bodies. The movement of the screws is transmitted by the interaction between the screw particles and the fluid particles, and a different distribution of the fluid particles is finally formed according to the distance between the fluid particles and the screw particles together with the varying geometry of the flow channel.

### 2.6. Computational Time

The present study is based on the open-source SPH solver DualSPHysics v5.0.1 [47] (www.dual.sphysics.org), and the simulation is performed with 32 Intel XEON cores (2.1 GHz), an NVIDIA Tesla k40 GPU card, and 64GB RAM. As described in Table 2, there are four cases to be simulated for the whole study; one case for the fully filled state needs about 95 h to compute, and in the half-filled state, the time reduces to 50 h because the number of particles in the latter is half of that in the former. As a whole, it takes about 290 h to compute the four cases listed in Table 2.

## 3. Results and Discussion

As depicted in Figure 1, the plasticization unit for polymer processing is generally split into three parts: the solid conveying zone, the melting zone and the metering zone. If divided by the filled state for the above three parts, there are two states: the fully filled zone usually in the front of the unit and the partially filled zone mostly in the back of the unit. Therefore, the rotational speed excitation superimposed on the screw would affect both states in the whole flow channel. And the focus of this paper is to analyze how much the vibration field affects the flow property. At present, all parameters that can be obtained from computation by the SPH software DualSPHysics are velocity, pressure, and particle distribution. Therefore, the following content presents the results and corresponding discussions mainly from the above aspects.

### 3.1. Fully Filled State

**Figure 3:**
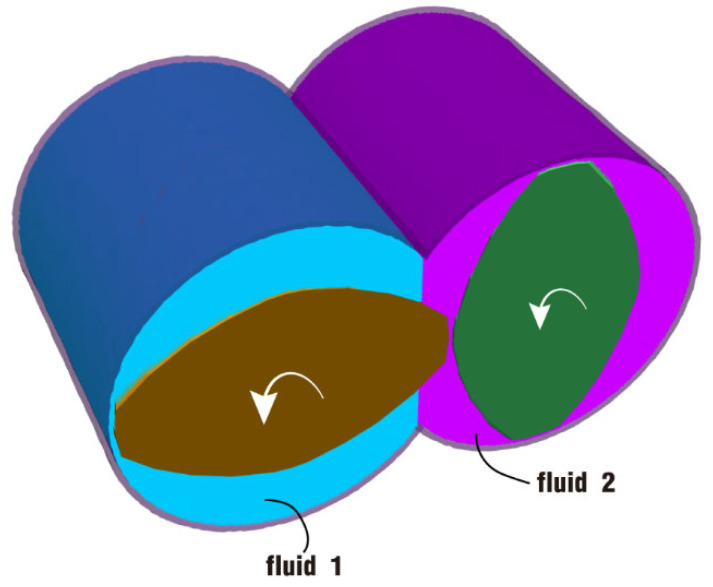
Initial setup of the fully filled state for case 1 and case 2.

To begin with, let us discuss the fully filled state. Initially, the channel is equally split into two parts: the left part (marked in blue) is fluid 1 and the right part (marked in purple) is fluid 2, as shown in Figure 3. The twin screws are loaded by the same rotation speed, counter-clockwise and simultaneously. And the barrel is transparent in the figure, mainly for the convenience of displaying the internal fluids. This is the original state at the beginning of the simulation.

#### 3.1.1. Velocity Distribution

**Figure 4:**
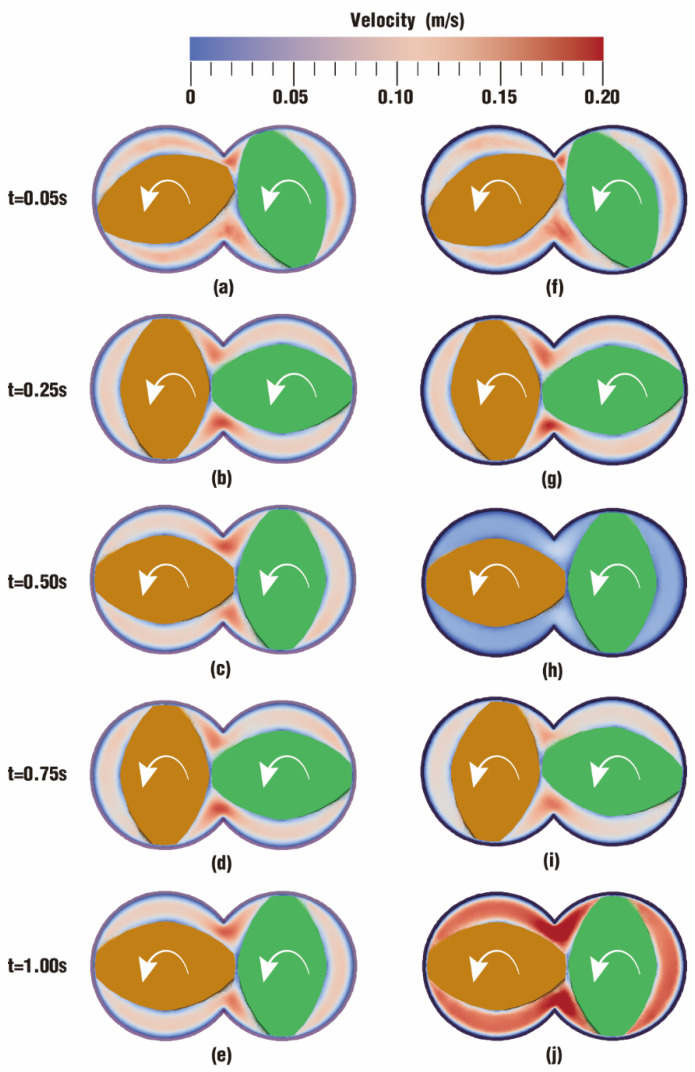
Velocity distribution at certain timesteps over a rotation: (**a**–**e**) results of case 1; (**f**–**j**) results of case 2.

The velocity distribution directly displays the movement situation of particles in the flow field, and it can also be an important metric to judge whether one processing method is more suitable for polymer materials than other methods. Figure 4 describes the comparison of velocity distribution contours at certain timesteps over one rotation between case 1 and case 2. The color in the channel corresponds to different velocities. In order to clearly display the velocity distribution for both case 1 and case 2, the color scale is selected from 0 m/s (denoted as blue) to 0.20 m/s (denoted as red) even though there are velocities greater than 0.20 m/s at some moments.

According to the figure, it can be seen that the overall velocities in the intermeshing zone in the two cases are always larger than those in the non-meshing zone, which is a result of the strong shear and tensile forces caused by the changing chamber between the twin screws. In addition, due to wall adhesion, velocities along the outer surface of the twin screws and the inner surface of the barrel are colored in light blue, indicating a very low value of velocity.

From Figure 4, it can also be found that there are some differences in the velocity distribution between the two cases. By contrast, the velocities in case 1 evolve much more stably than those in case 2. Typically, at the same timestep t = 0.50 s, the velocity distribution in Figure 4h is rather lower than that in Figure 4c, while the velocities in Figure 4j become much higher than those in Figure 4e at time t = 1.00 s. There is only one difference in the parameter settings for case 1 and case 2 in Table 2, that is, case 1 rotates at a constant speed while case 2 rotates at a constant speed superimposed with an excitation speed. Obviously, it can be inferred that the superimposed excitation takes effect in the above difference.

**Figure 5:**
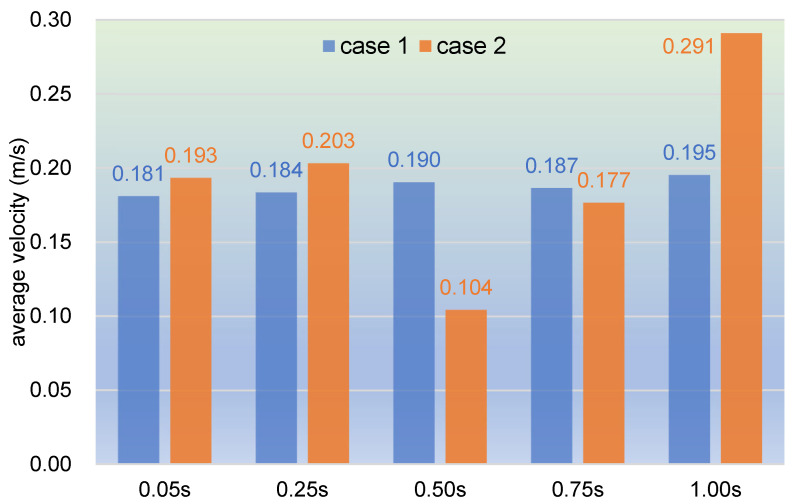
Average velocity of selected timesteps over the whole simulation.

In order to study quantitatively, the velocities of the top 1000 particles are collected and averaged at each timestep listed in Figure 4 for both case 1 and case 2, as shown in Figure 5. The chart reveals that the lowest average velocity is in case 2 at 0.50 s with 0.104 m/s, while the highest average velocity occurs in case 2 at 1.00 s with 0.291 m/s. The rest of the timesteps do not show a large variation in average velocity, and the range is between 0.177 m/s and 0.203 m/s. It can also be seen that average velocity in case 2 increases very slightly at 0.05 s and 0.25 s, from 0.193 m/s to 0.203 m/s, then drops sharply to 0.104 m/s at 0.50s, and goes up till the maximum 0.291 m/s at 1.00 s. In contrast, the average velocity in case 1 changes quite stably, varying between 0.181 and 0.195 m/s. These results are in good agreement with those acquired from Figure 4.

**Figure 6:**
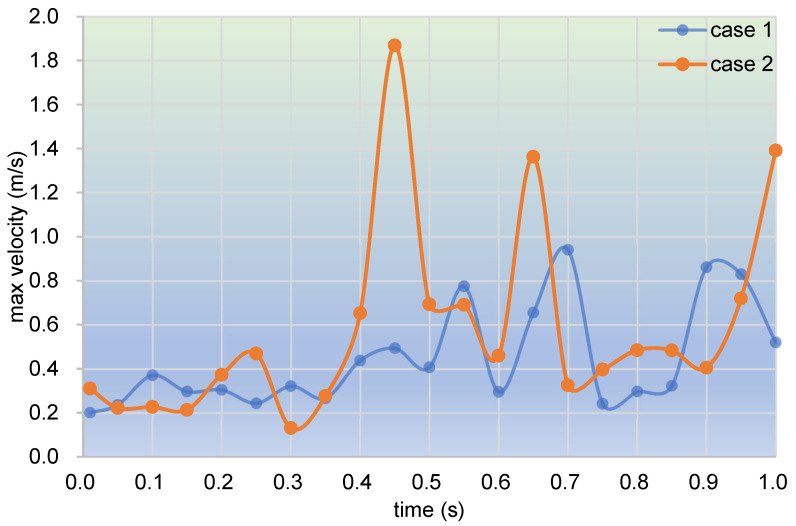
Change in max velocity versus time during one second.

Figure 6 depicts the evolution of max velocity every ten timesteps over the whole simulation time for case 1 and case 2. It can be seen from the figure that the max velocity for both case 1 and case 2 takes on a concussion change within the simulation time. Specifically, within the first 0.4 s, the max velocities mostly range between 0.15 and 0.45 m/s, and the concussion begins to enlarge sharply after 0.4 s. The max velocities of case 1 lie within 0.2–0.8 m/s, whereas those of case 2 fluctuate from 0.3 m/s up to about 1.9 m/s. The fluctuation range of velocities in case 2 is about 2.5 times larger than that in case 1. In summary, case 2 has a larger concussion amplitude than case 1 as a result of the pulsation excitation.

**Figure 7:**
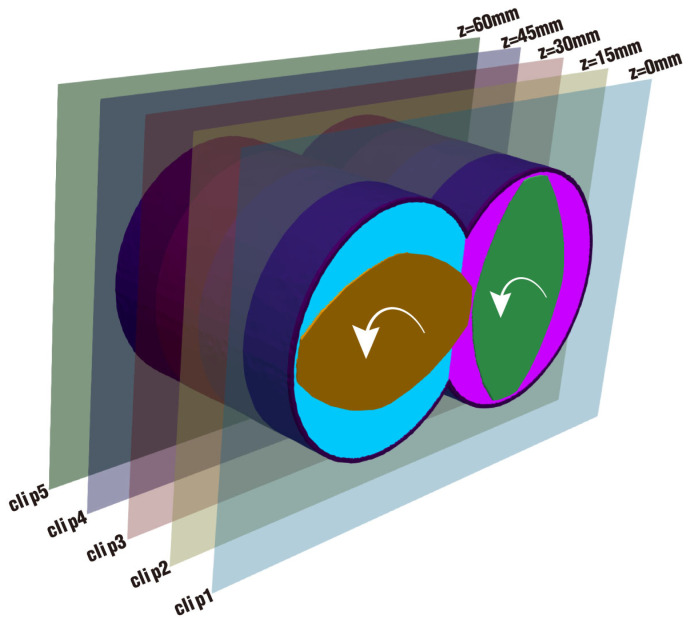
Five clips established perpendicular to the Z axis.

To improve the visualization of the velocity distribution inside the channel for the twin screw component, five clips with an equal spacing of 15 mm are established from the inlet to the outlet, which are along the Z axis of the flow field, as displayed in Figure 7. As is known, the flow field is composed of numerous particles in the SPH simulation by DualSPHysics. Too few particles in each clip may lead to too much blank and clearance, while too many particles would take up a larger space, which makes the clip meaningless. After detailed trials, the thickness of 5 mm is found to be suitable for one clip to collect enough particles for a clear image.

**Figure 8:**
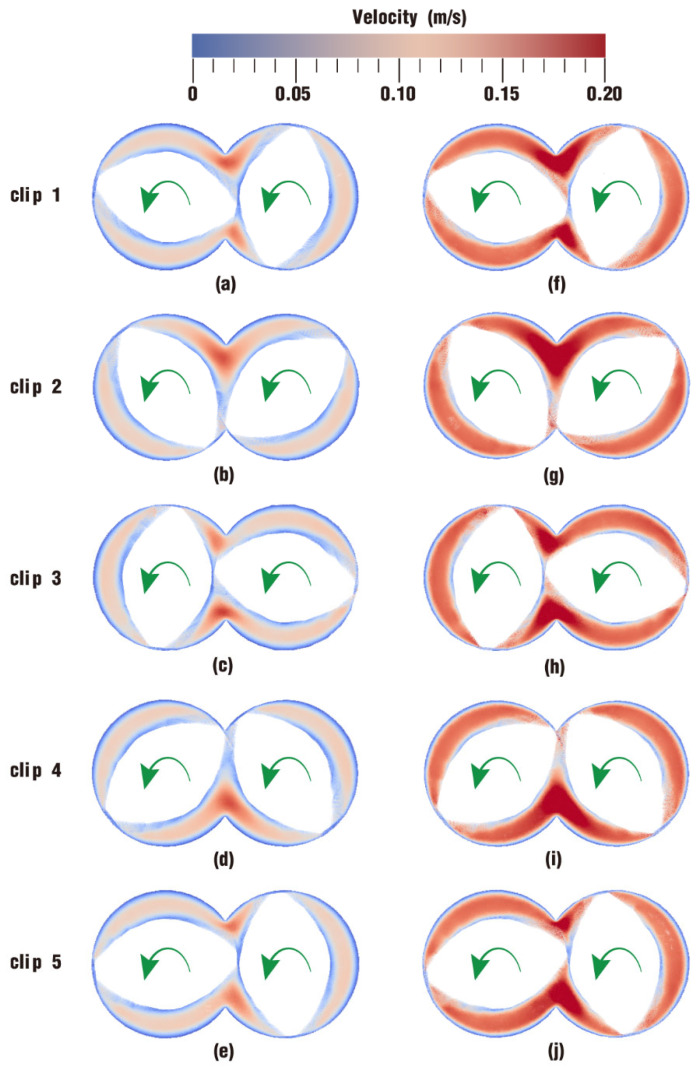
Velocity distribution on the clips after one rotation: (**a**–**e**) results of case 1; (**f**–**j**) results of case 2.

Detailed results of the velocity distribution contours on the five clips shown in Figure 7 at t = 1.00 s are presented in Figure 8. For better observation, the barrel and the twin screws are not shown. On the whole, the velocity distribution in the intermeshing zone and near the boundary surfaces shown in Figure 8 is quite similar to that in Figure 4. A slight difference is that in Figure 8, there are higher velocities in the intermeshing region and approximately zero velocities in the boundary surface region. In Figure 8, there is a distinct difference that velocities on all the five clips in Figure 8f–j are much greater than those in Figure 8a–e. Consequently, it can be concluded that the whole flow field in case 2 acquires a relatively high velocity as a result of the introduction of the rotational speed vibration excitation.

**Figure 9:**
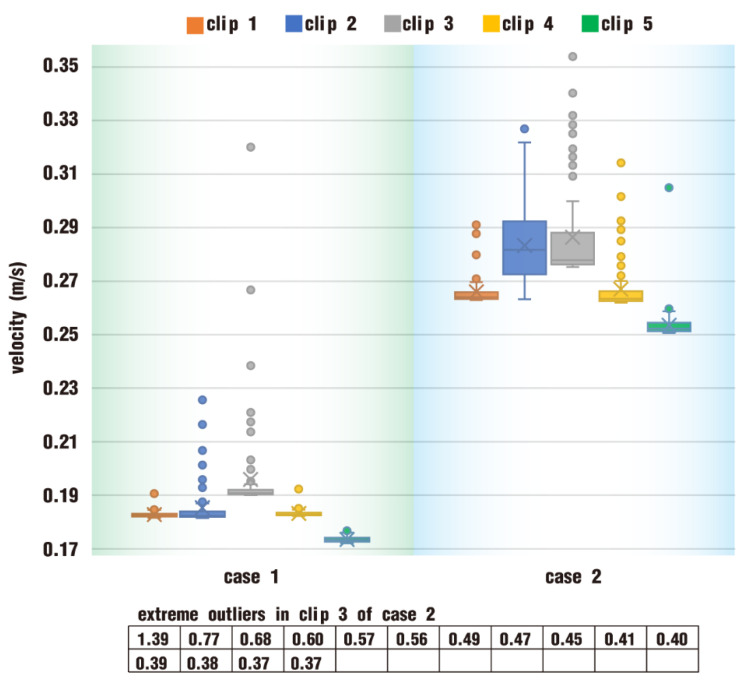
Statistics of top 100 velocities in five clips after one rotation.

In order to conduct a quantitative analysis of velocities in the clips, the top 100 velocities are gathered to compile statistics, as shown in Figure 9. It can be suggested that the box plot, either in case 1 or in case 2, indicates that velocities in clip 1, clip 2, clip 4, and clip 5 all take on a centralized data distribution. Nevertheless, velocities on clip 3 for both case 1 and case 2 have many outliers. The difference lies in that the outliers in clip 3 of case 1 are a little mild, while those in clip 3 of case 2 are more extreme, many of which are separately listed in descending order at the bottom of the figure. Generally speaking, the values of the median, the average (denoted as X), and the maximum in case 2 are much greater than those in case 1, thus allowing the particles in case 2 larger shearing and stretching effects along the flow channel. This illustrates that the superposition of a vibration field on the rotation of twin screws has an important impact on the flow property of fluids.

**Figure 10:**
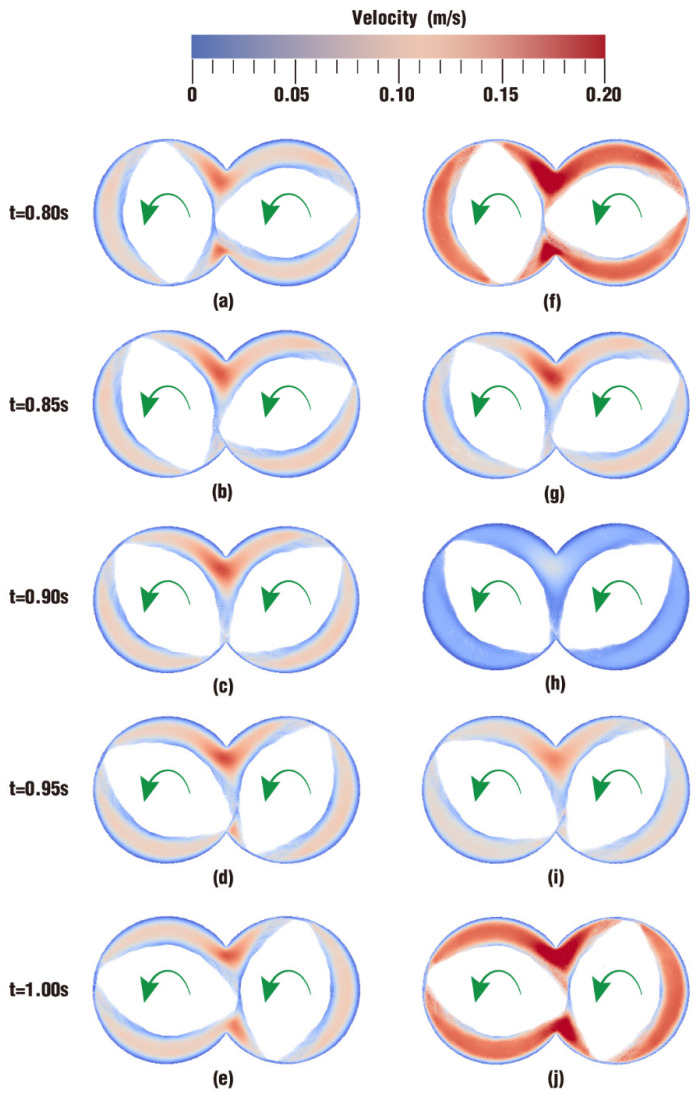
Velocity distribution on clip 1 within the last period: (**a**–**e**) results of case 1; (**f**–**j**) results of case 2.

The following particularly focuses on only one period of the simulation on one clip. As introduced in Section 2.4, the frequency of vibration excitation is 5 Hz. In other words, the period of the excitation is 0.2 s and there are five complete periods within 1.00 s. Here, the last period is selected to analyze the final effect of the superimposed excitation on the velocity distribution. Figure 10 shows the velocity contours of case 1 and case 2 on clip 1 every 0.05 s in the last period. Obviously, Figure 10f,j are much redder than Figure 10a,e, indicating that the former two timesteps have a bigger velocity; Figure 10h is rather bluer than Figure 10c, indicating the former has a smaller velocity. And in the final two pairs of figures, there is only a little color difference, which implies that the two pairs have similar velocity values. Hence, these results suggest that the velocity in case 2 varies back and forth from big to small, presenting an oscillatory change. This oscillatory change in velocities is conducive to changing the flow state for the material particles in the flow channel. The position change of the material particles plays a great role in improving the mixing effect of the particles.

**Figure 11:**
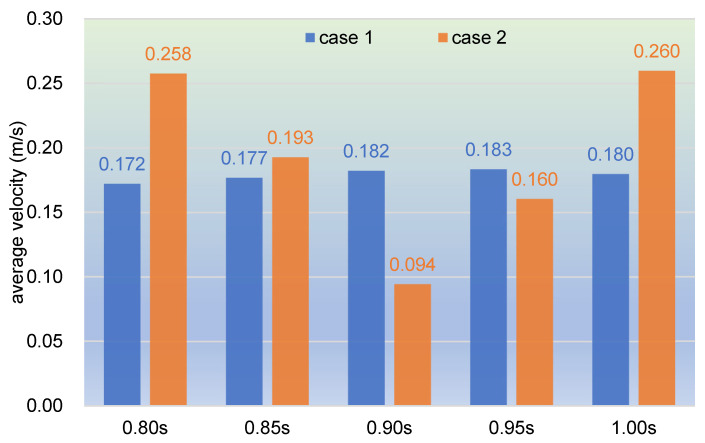
Average velocity of selected timesteps within the last period.

As used in Figure 5, we select the 1000 particles with the top velocities in clip 1 to calculate the average velocity for the sake of representing the flow property in a quantitative analysis, as shown in Figure 11. It can be observed from the bar chart that the average velocity rises rapidly from case 1 to case 2 both at 0.80 s and at 1.00 s, from 0.172 m/s to 0.258 m/s and from 0.180 m/s to 0.260 m/s, respectively. However, at 0.90 s, the average velocity drops sharply from case 1 to case 2, from 0.182 m/s to 0.094 m/s. And at the other two timesteps, 0.85 and 0.95 s, the average velocity changes slightly up and down. Overall, the average velocity for timesteps in case 1 changes very mildly, while the value in case 2 varies significantly, which is quite consistent with the results shown in Figure 10.

**Figure 12:**
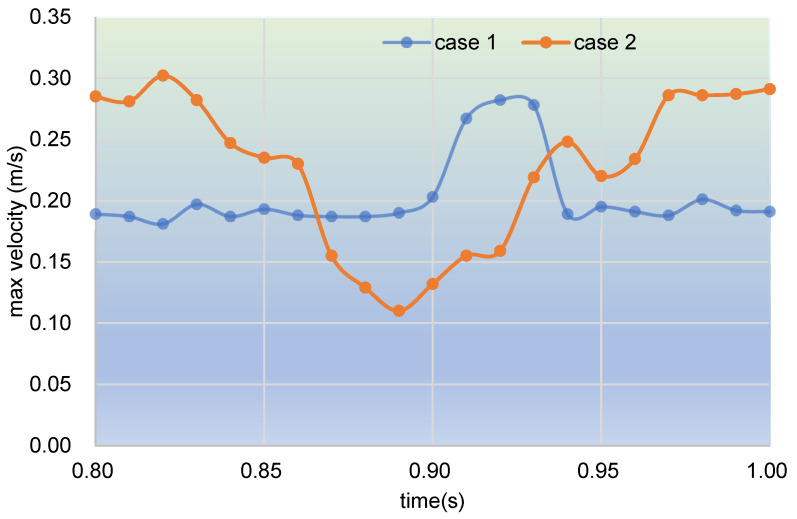
Time evolution of max velocity on clip 1 during the last period.

Figure 12 analyzes the time evolution of max velocity on clip 1 during the last period. As shown in the graph, about two-thirds of the max velocity curve of case 2 is above the curve of case 1, which happens in the beginning and ending parts of the last period. In contrast, the max velocity in the middle part of case 2 is below that of case 1. These results are in good agreement with those shown in Figure 10.

From the above analysis of velocity either in the whole channel or on clips along the channel, it can be concluded that the sinusoidal pulsation from rotational speed can lead to a varying velocity field against the steady velocity field without any excitation. All in all, no matter whether it is in only one period or in the whole simulation time, this varying velocity field can be obtained. Moreover, the varying field could allow the particles to have a larger average velocity.

#### 3.1.2. Particle Distribution and Pressure

**Figure 13:**
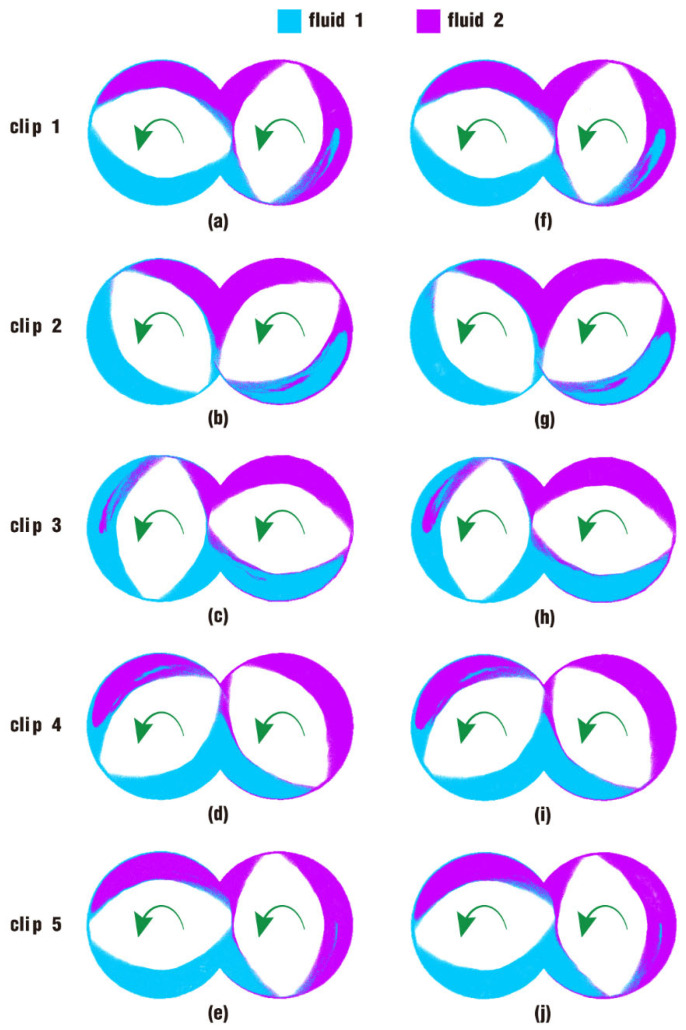
Particle distribution on clips after one rotation: (**a**–**e**) results of case 1; (**f**–**j**) results of case 2.

After seeing the velocity distribution for case 1 and case 2, let us now move on to the particle distribution, which is a direct representation of the velocity distribution. For fully filled states like case 1 and case 2, the particle distribution can be more clearly observed if it is inside the flow channel. Therefore, here, snapshots are obtained only on clips built in Figure 7, with results at t = 1.00 s shown in Figure 13. On the whole, it can be seen that there is no significant difference in the flow distribution of particles between states in case 1 and case 2. Just a few little differences can be found in local areas. For example, in the lower right region of both Figure 13a,f on clip 1, which was originally the location of fluid 2, there are more particles from fluid 1 in Figure 13f than in Figure 13a. Similarly, in the upper left region of both Figure 13c,h on clip 3, which was originally the location of fluid 1, there are more particles from fluid 2 in Figure 13h than in Figure 13c.

**Figure 14:**
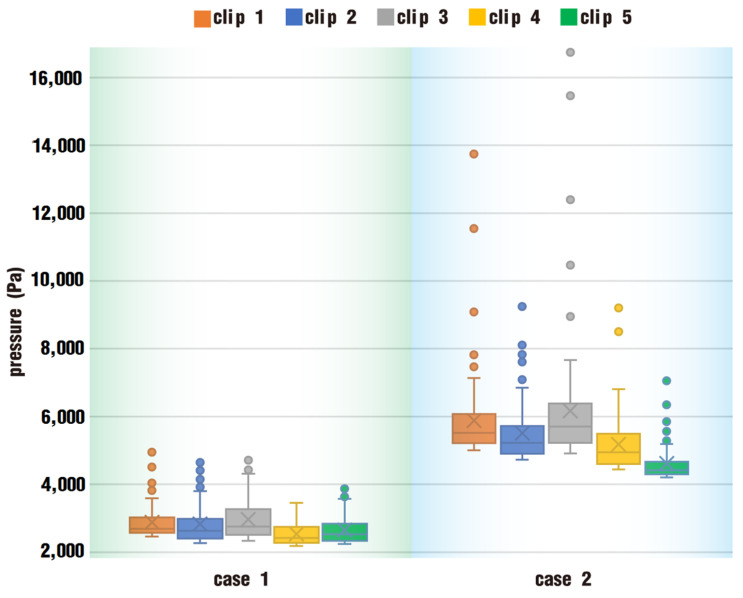
Statistics of top 100 pressures in five clips after one rotation.

As is known, the twin screws constitute the rotating dynamic boundaries, which build pressure in the flow channel and produce the pressure flow. Obviously, the distribution of particles is affected by the pressure produced from the rotation of the twin screws. Next, let us have a look at the pressure on the above clips. Here, we select the top 100 pressures of particles in each clip and draw a box plot, as shown in Figure 14. As a whole, the bodies of the five box plots in case 1 are between 2000 and 4000 Pa, while those in case 2 are between 4000 and 8000 Pa. The latter is almost twice as large as the former, indicating that particles in case 2 generally experience much higher pressure than those in case 1.

Additionally, each clip in case 2 has more outliers than its counterpart in case 1, and outlier values in case 2 are more discretely scattered in a large range. Specifically, outlier pressures in clip 3 of case 2 vary from 9000 up to 16,500 Pa. In contrast, those in case 1 are rather milder, changing from 3800 to 5200 Pa.

**Figure 15:**
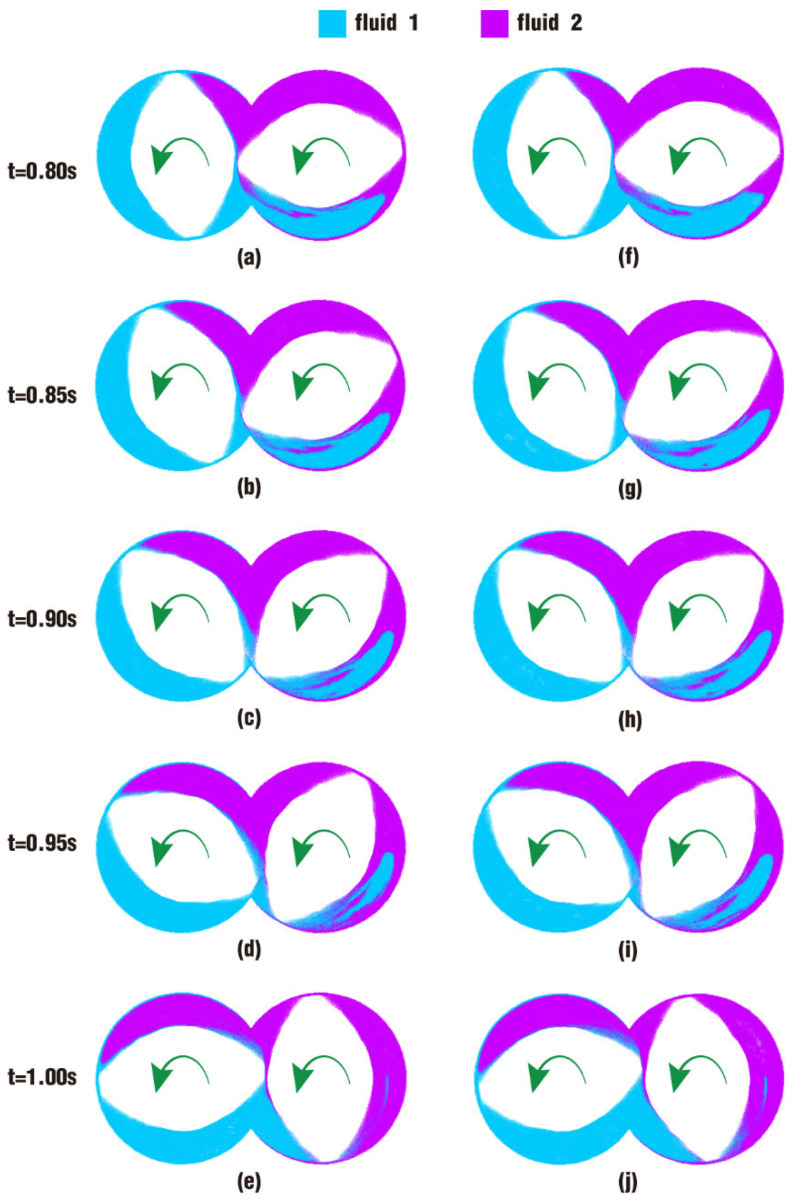
Particle distribution on clip 1 within the last period: (**a**–**e**) results of case 1; (**f**–**j**) results of case 2.

A comparison of results for particle distribution contours on clip 1 during the last period of the simulation time is shown in Figure 15. It can be seen from the figure that the interface between fluid 1 and fluid 2 develops as time goes on. At first glance, the distribution contours in Figure 15 appear to be similar to those in Figure 13, and the difference in particle distribution between the two cases at the same time can hardly be recognized without carefulness. In the bottom right areas of Figure 15a,f at 0.80 s, and Figure 15b,g at 0.85 s, there are a few more particles from fluid 2 in the blue area in the latter figures than in the former figures. And it is apparent that there are more particles from fluid 1 in the bottom right region in Figure 13i than in Figure 13d, which is initially set for fluid 2.

By comparing the results from Figure 14 and Figure 15, the pressure in case 2 is larger than that in case 1, and the particle distribution is a little better in case 2 than that in case 1. As a consequence, we can learn that the pressure in the channel for case 2 plays an important part in molding the shapes and sizes of its particle distribution.

**Figure 16:**
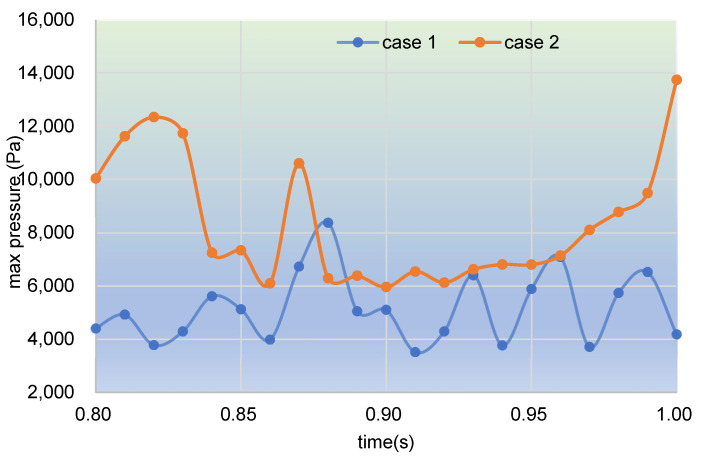
Time evolution of max pressure on clip 1 during the last period.

Figure 16 covers the time evolution of the max pressure on clip 1 within the last period. It can be found that the pressure changes up and down no matter whether it is in case 1 or in case 2. In other words, at the same place (clip 1) in the channel, the pressure is not unchangeable. In case 1, most pressures of particles are between 4000 and 7000 Pa, while those in case 2 are between 6000 and 12,000 Pa. Clearly, in the last period, particles in case 2 suffer much larger pressure than those in case 1.

**Figure 17:**
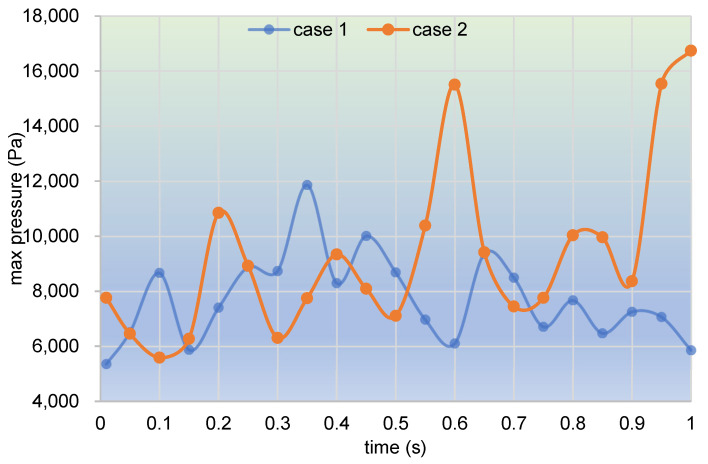
Time evolution of max pressure in the whole channel within the simulation time.

Following the above analysis, values of max pressure changing with time in the whole channel are counted and plotted, as shown in Figure 17. It can be clearly seen that the max pressure curve in case 1 first increases up to 12,000 Pa at about 0.35 s, and then gradually drops down for the rest of the time. The max pressure curve in case 2, however, fluctuates upwards for the whole simulation time. The two curves are neck and neck before the time 0.5 s, while the latter rockets up to about 17,000 Pa at 1.00 s, which is far beyond the value of 6000 Pa of the former. Note that the only difference in settings between case 1 and case 2 is that case 1 is without excitation and case 2 is under excitation. Thus, it can be indicated that the vibration excitation superimposed in case 2 plays an important part in improving the pressures in the flow field.

From the above results and discussion, it can be concluded that for the fully filled state, whether it is from the perspective of the overall flow field or the internal slice data, or from the view of time evolution, the superimposed velocity pulsation field can indeed produce a pulsating change in velocity and pressure inside the flow channel. Moreover, the average values of physical quantities in this field are larger than the corresponding values obtained under the condition of no excitation field, which is very conducive to the complete mixing of the material particles in the flow channel. The reason is that different from the steady movement of the constant speed, a change in the physical field such as the speed generated by the excitation field causes the material particles in the flow channel to produce a certain drag and impact effect. These conclusions prove that the pulsating velocity field could play an important role in improving the mixing efficiency of the twin screw extruders.

### 3.2. Partially Filled State

**Figure 18:**
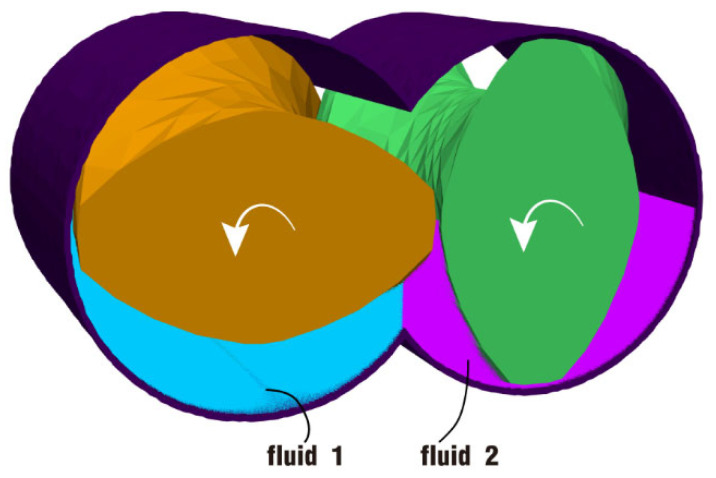
Initial setup of the half-filled state for case 3 and case 4.

In this part, the partially filled state is analyzed in two cases: case 3 without rotation excitation and case 4 under rotation excitation, as illustrated in Figure 18. Contrary to the fully filled state, the channel here is half filled by particles. In the same way, the fluid is also equally split into two parts: the lower left part (marked in blue) is fluid 1 and the lower right part (marked in purple) is fluid 2. The twin screws are also loaded by the same rotation speed, counter-clockwise and simultaneously. And this is the original state at the beginning of the simulation.

#### 3.2.1. Velocity Distribution

**Figure 19:**
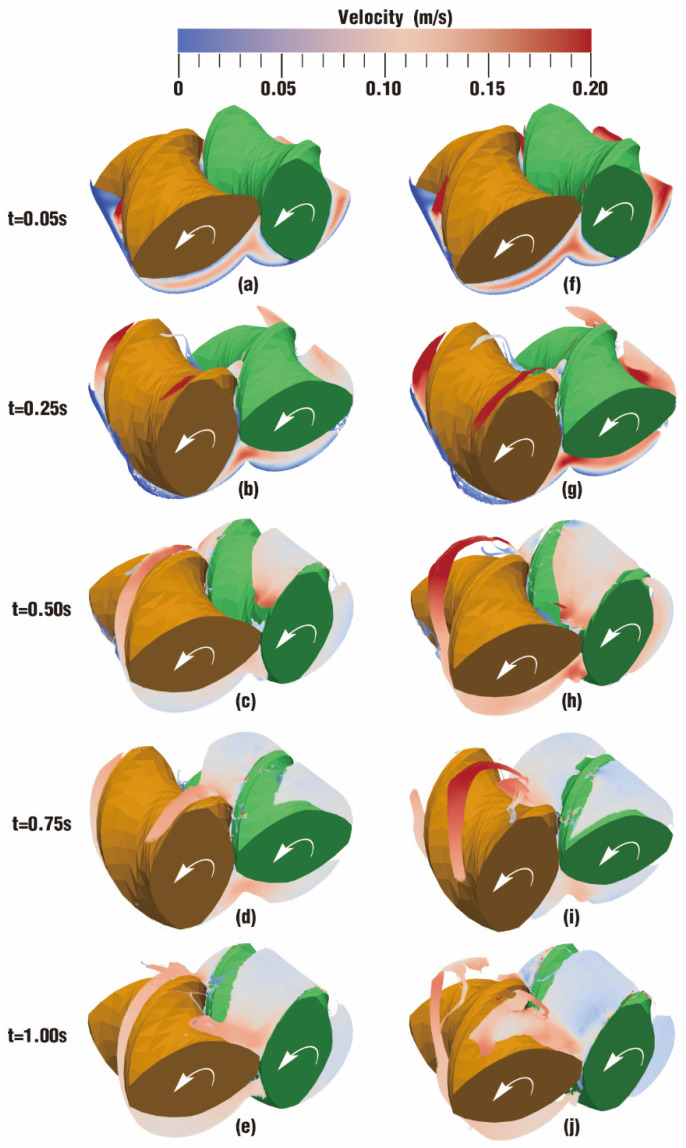
Velocity distribution at certain timesteps over a rotation: (**a**–**e**) results of case 3; (**f**–**j**) results of case 4.

A comparison of results for the velocity distribution contours at certain timesteps within a rotation of the twin screws is depicted in Figure 19. It can be seen that the contour color gradually fades both in case 3 and case 4 as time goes on, implying that velocities are dropping. Moreover, there is a decided difference in color between case 3 and case 4. Most areas of velocity contours in case 4 are much redder than those in case 3, indicating that velocities in the former are larger than those in the latter. Specifically, there are more particles colored in red in the nip regions and screw flights in Figure 19g,h than in Figure 19b,c.

It is worth noting that particles in case 3 and case 4 perform a free surface flow, which is quite different from the fully filled state in case 1 and case 2. As stated in Figure 18, the channel in case 3 and case 4 is partially filled, leaving a large space for the fluid to flow freely. Therefore, there are very few particles advancing into the gaps between the twin screws and the barrel, and most particles flow forward along the screw channel.

**Figure 20:**
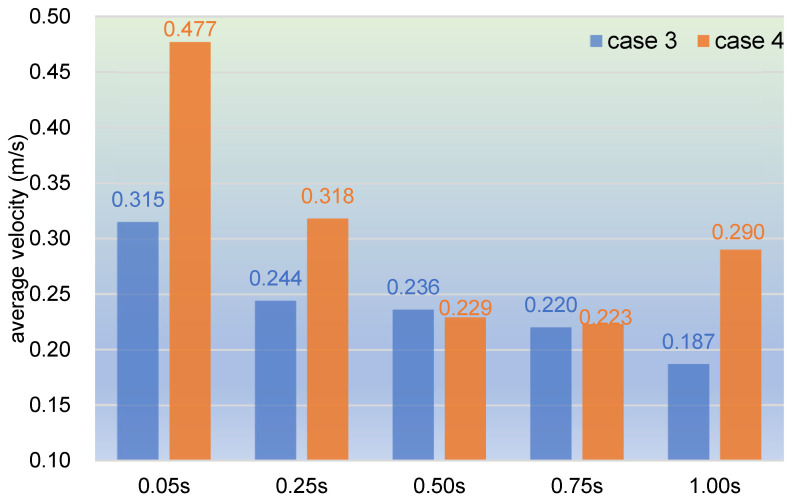
Average velocity of selected timesteps corresponding to Figure 19.

As used in Figure 5, here, we select the 1000 top velocities of particles at selected timesteps corresponding to Figure 19 to calculate the average velocity to represent the flow property, as displayed in Figure 20. It can be observed from the bar chart that the average velocity in case 3 decreases gradually till the end of the timesteps, from 0.315 m/s at 0.05 s to 0.187 m/s at 1.00 s. And the average velocity in case 4 drops rapidly from 0.477 m/s at 0.05 s to 0.229 m/s at 0.50 s, then slightly slips to 0.223 m/s at 0.75 s, and finally rises upward to 0.290 m/s at 1.00 s. As a whole, the changing trend is basically in agreement with the results obtained in Figure 19. By contrast, velocities in case 3 vary very mildly with a fluctuation range of [0.187, 0.315], while those in case 4 change remarkably with a range of [0.223, 0.477]. It can also be found that at the timesteps 0.05 s, 0.25 s, and 1.00 s, the average velocities in case 4 are all greater than those in case 3, and in the final two timesteps, the average velocities in the two cases are almost the same. Therefore, particles in case 4 possess a larger velocity than those in case 3, which is quite consistent with the results shown in Figure 19.

**Figure 21:**
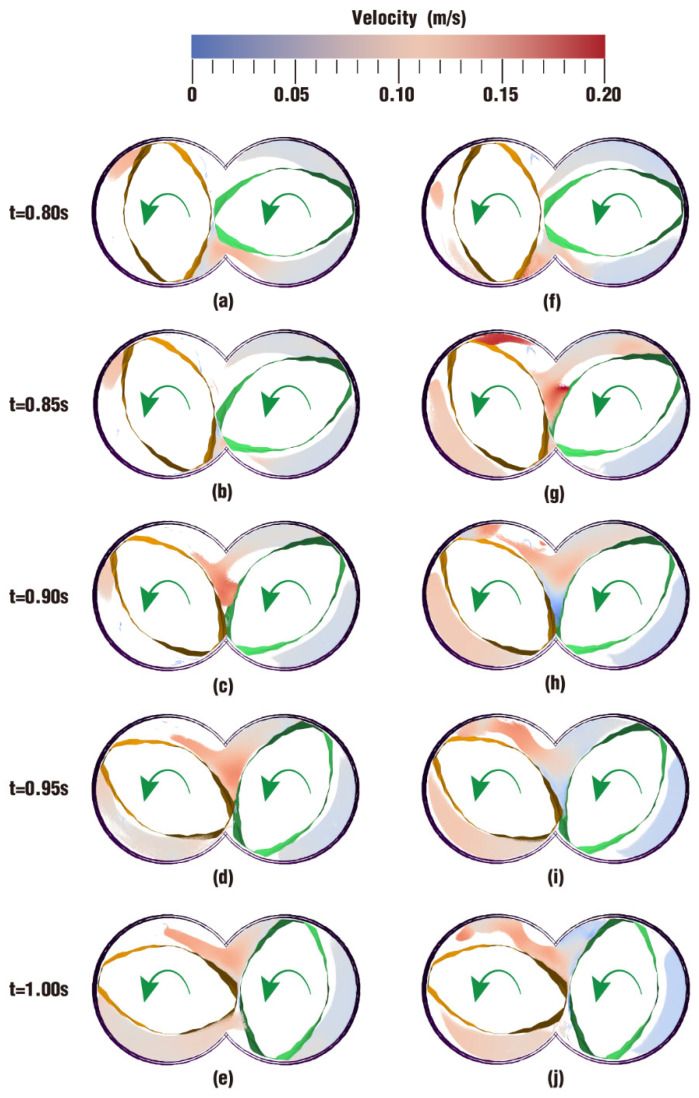
Velocity distribution on clip 1 within the last period: (**a**–**e**) results of case 3; (**f**–**j**) results of case 4.

Next is an inside view of clip 1 (the same position as shown in Figure 7). Figure 21 compares the velocity distribution contours on clip 1 within the last period between case 3 and case 4. It can be seen that at 0.85 s, Figure 21g shows larger velocities in the intermeshing region and the upper left region than Figure 21b does. Other than this timestep, no significant difference can be found between case 3 and case 4.

However, there is a disparate phenomenon in Figure 21 that velocities change in a steady way, which is quite different from the results obtained in Figure 10 in fully filled cases. In Figure 10, velocities in case 2 present an oscillatory change against those in case 1, while in Figure 21, velocities take on a relatively mild change no matter whether they are in case 3 or case 4.

**Figure 22:**
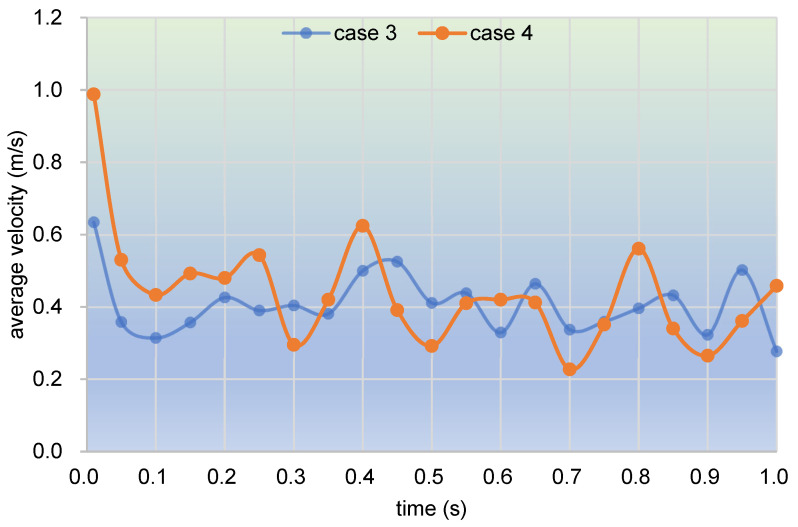
Time evolution of average velocity in the whole channel within one second.

Considering the special property of free surface flows in the partially filled state, here, we collect and count the average velocities of the top 100 particles in the whole channel within one second (corresponding to one rotation), as demonstrated in Figure 22. It can be seen that average velocities in both case 3 and case 4 take on a decreasing concussion change during the one second. At the beginning, both cases drop sharply and then oscillate downward till the end. To be specific, the average velocities in case 3 vary roughly between 0.3 m/s and 0.5 m/s, while those in case 4 range mostly from 0.2 m/s to 0.6 m/s. Thus, by and large, these trends suggest that the average velocities in case 4 have a larger fluctuating range than those in case 3.

#### 3.2.2. Particle Distribution and Pressure

**Figure 23:**
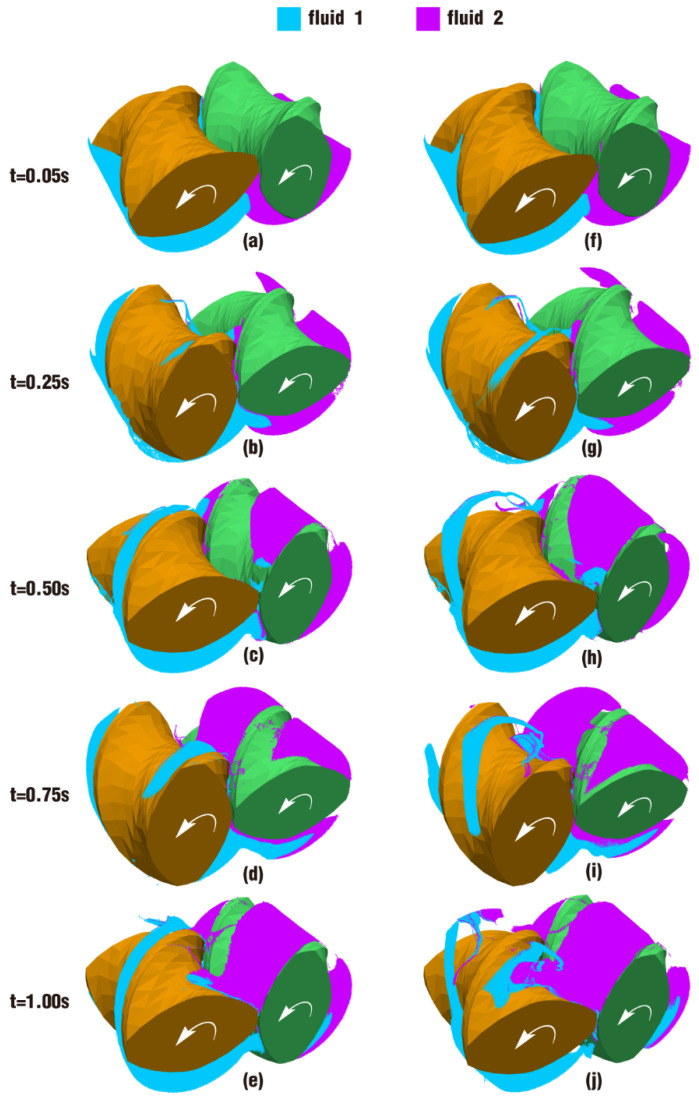
Particle distribution at certain timesteps over a rotation: (**a**–**e**) results of case 3; (**f**–**j**) results of case 4.

The results for the particle distribution contours at certain timesteps within a rotation for case 3 and case 4 are shown in Figure 23. It can be seen that in both cases, fluid 1 and fluid 2 are basically proceeding along the screw channel. Only very few particles run across the screw flight or into the gap between the twin screws. This is mainly because the free surface flow holds much freedom for fluids to move forward in the direction with the least resistance. Gaps between the twin screws and the barrel have greater resistance than the channel. Correspondingly, there are many more particles flowing along the channel. This is quite different from the fully filled state, in which the fluids move forward not only due to the dragging of the screws but also due to the squeezing of the pressure resulting from the rotation of the screws.

**Figure 24:**
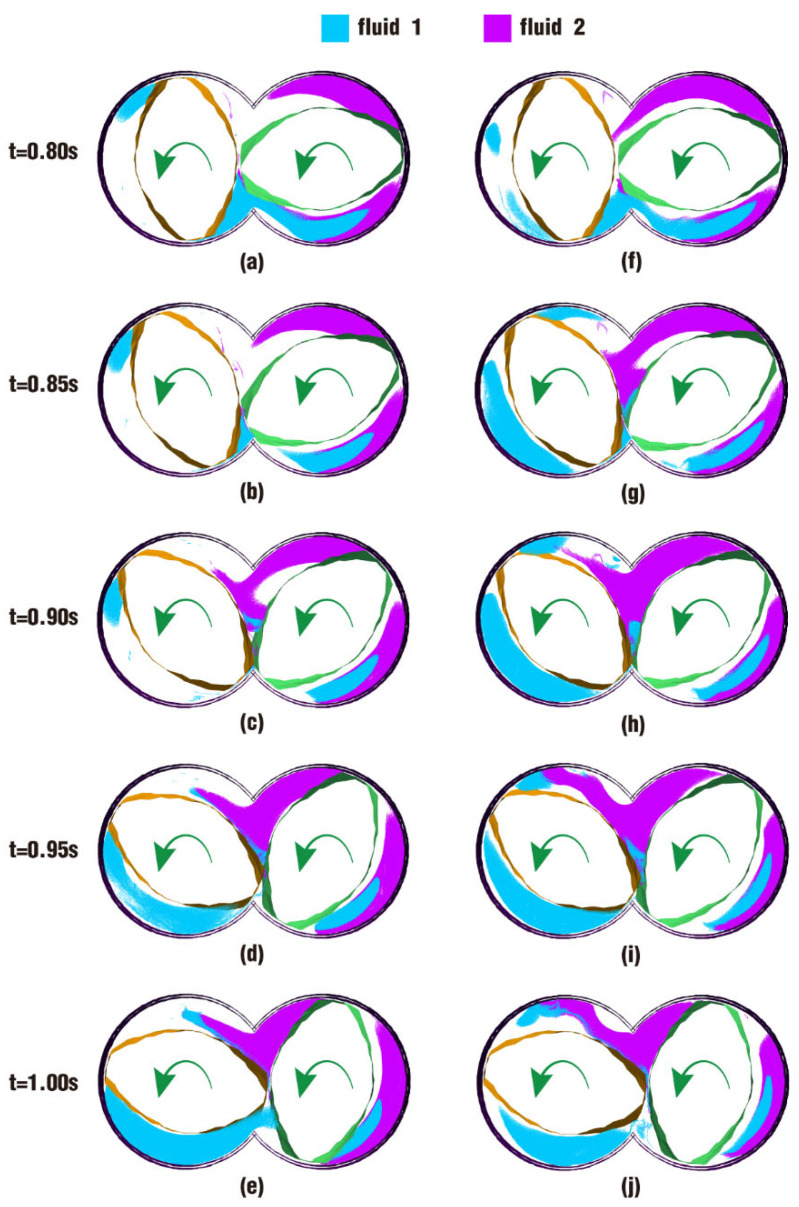
Particle distribution on clip 1 within the last period: (**a**–**e**) results of case 3; (**f**–**j**) results of case 4.

Through careful comparison of the contours between case 3 and case 4 in Figure 23, it can also be found that with the mixing process, the number of particles in the flow channels in Figure 24f–j is significantly higher than their counterparts in Figure 24a–e. In particular, the contrast between Figure 24j and 24e is much more obvious. In Figure 24j, the particles of fluid 2 almost fill the flow channel of the screw at its position, and some particles even move into the flow channel of the matching screw. Similar to the previous analysis, it can be seen from the parameter settings of case 3 and case 4 in Table 2 that the only difference between the two cases is that case 4 has a velocity pulsation excitation. Therefore, it can be inferred that the velocity pulsation excitation is beneficial to promoting the motion of particles, so as to achieve a better distribution mixing effect.

The above phenomenon can be more clearly seen from a cross-section perpendicular to the Z direction of the channel, as shown in Figure 24. Figure 24 reveals the particle distribution contours on clip 1 within the last period. On the upper side of Figure 24f, the front particles of fluid 2 arrive at the intermeshing zone, then gather into a group in Figure 24g. When the number of particles there reaches a certain value, particles begin to bypass the gap between the twin screws and climb up along the left screw channel, as shown in Figure 24h–j. It is the same in Figure 24a–e, and the only difference is that the whole process occurs several timesteps later than in Figure 24f–j. Moreover, from Figure 23i,j against Figure 23d,e, and Figure 24f–j against Figure 24a–e, it can be deduced that particles in case 4 have a higher displacement and number than those in case 3, giving the two fluids in the former a better mixing effect.

**Figure 25:**
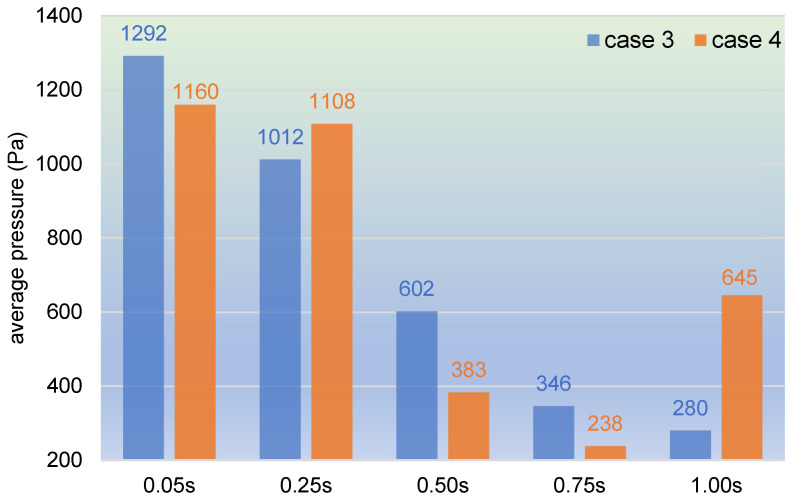
Average pressure of selected timesteps within one second.

In Figure 25, 1000 particles of top velocities are selected at several timesteps to acquire the average pressure to study the flow property. It can be seen from the bar chart that as a whole, the changing trend is basically downward for both case 3 and case 4. At the beginning of the simulation, there are high pressures in both cases, and then the pressures start to drop straight to one-fourth of the original values. Specifically, the average pressure in case 3 decreases rapidly till the end of the simulation time, from 1292 Pa at 0.05 s to 280 Pa at 1.00 s, reducing by approximately 78%. And the average pressure in case 4 slips slightly from 1160 Pa at 0.05 s to 1108 Pa at 0.25 s, then drops sharply to 383 Pa at 0.50 s and to 238 Pa at 0.75 s, and finally rises upward to 645 Pa at 1.00 s.

**Figure 26:**
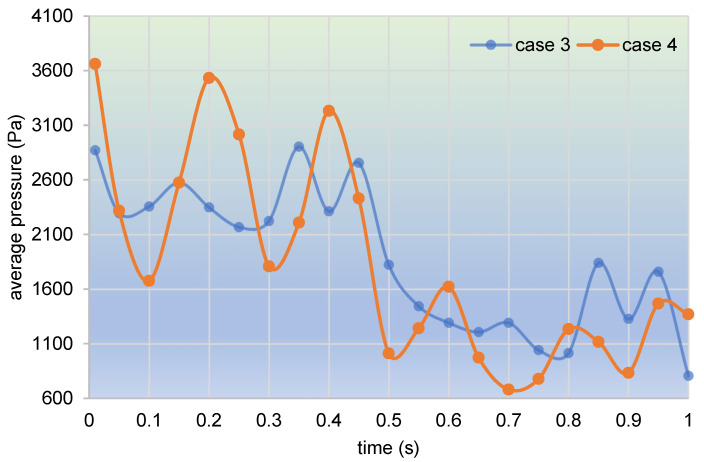
Time evolution of average pressure in the whole channel within one second.

Taking into consideration a smaller time interval for the sake of more details, here, we collect and count the average pressure values of the top 100 particles in the whole channel within one second, as plotted in Figure 26. It can be seen that average pressures in both case 3 and case 4 take on a downward oscillating trend during the one second. There is an apparent time tipping point, that is, at 0.5 s. Within the first 0.5 s, average pressures in both case 3 and case 4 oscillate around a pressure value of 2500 Pa. Then, at a time near 0.5 s, the values of average pressure undergo a sharp drop. And in the last 0.5 s, the average pressures change around the value of 1200 Pa. Obviously, the oscillation amplitude of average pressures in case 4 is approximately between 1600 and 3600 Pa in the first 0.5 s, and between 600 and 1600 Pa in the last 0.5 s. And that value in case 3 is approximately between 2100 and 2900 Pa, and between 800 and 1800 Pa, respectively. Therefore, it is clear that on the whole, particles in case 4 have a larger oscillation amplitude than those in case 3.

From the above results and discussion for the partially filled state, it can be concluded that no matter whether it is from the perspective of the overall flow field, the internal slice data, or from the view of time evolution, the superimposed velocity pulsation field can also lead to a pulsating change in velocity and pressure inside the flow channel as in the fully filled state. Similarly, the average values of physical quantities in the pulsating condition are greater than those in the vibration-free condition. It is worth noting that the partially filled flow is a kind of free surface flow, and the flow of fluid presents some new characteristics, that is, the physical quantities such as the velocity and the pressure gradually decrease with the mixing process.

Finally, the statistical results for max velocities and pressures under the operating conditions of four cases within the simulation time are summarized, as shown in Table 3. On the one hand, as can be seen from the table, for the fully filled state, the introduction of speed excitation can enhance the maximum velocity and pressure in the flow field, which increase by 30.1% and 19.8%, respectively. However, for the half-filled state, the introduction of the vibration field affects it in a different way. The maximum pressure in the flow field increases by 23.5%, while the maximum speed decreases by 10.4%. On the other hand, compared with the two cases in the fully filled state, the maximum velocity of the particles in the half-filled two cases increases by 75.8% and 21.1%, respectively, while their maximum pressure decreases by 30.5% and 28.3%, respectively. The reason, as mentioned above, is that the pressure field is easy to establish in the fully filled flow channel, and the movement of fluid particles is driven both by the drag of the twin screws and the pressure formed in the channel. By contrast, due to the existence of free space, the flow channel in the half-filled state cannot build a pressure field, and the movement of fluid particles is singly driven by the drag of the twin screws, which allows the particles to move freely along the curve of minimum resistance, which is known as a free surface flow.

In addition, as shown in Figure 1, there are usually three different areas for the material to go through from the inlet to the outlet: the conveying zone, the melting zone and the metering zone. The filling state of the channel is different in these three zones, which evolves from a partially filled state to a fully filled state. Together with the results from Table 3, the introduction of the pulsation vibrating field has different effects on the velocity and pressure for different filling states in the flow field. Therefore, it would be of great importance to balance the vibration parameters so as to obtain the best effect on the whole.

## 4. Conclusions and Future Work

In this study, a numerical investigation is performed to study the flow properties of fluids under a vibration excitation system. Specifically, a speed pulsation mechanism is introduced, and a sinusoidal rotational speed is loaded on the twin screws by using the meshless method SPH. Both the fully filled state and the partially filled state in a TSE are selected and analyzed in detail to determine the effect of superimposed excitation on the flow properties of polymers. The following conclusions are drawn:

(1) In the fully filled state, velocities in the intermeshing region are always larger than those in the non-meshing region as a result of the strong shear and tensile forces by the twin screws. The superimposed excitation can produce a changing velocity field, thus enhancing and weakening the flow field of polymers by turns. Meanwhile, the pulsation excitation can also form a much stronger pressure field, which is quite conducive to the flow of particles.
(2) In the partially filled state, particles tend to bypass the gaps between the twin screws and the barrel resulting from the free surface flow produced by the partially filled channel. The free surface leads to a decreasing trend in both the velocity field and the pressure field, which makes it quite different from the fully filled state. Even though the superimposed excitation cannot change the decreasing trend as a whole, it can strengthen the velocity and pressure fields to some extent.
(3) According to the statistical analysis results, the superimposed excitation loaded on twin screws can indeed change the physical fields, such as the velocity field and the pressure field, thus giving particles in the screw channel a more variable intensity of shearing and stretching action. Thus, there is a much better particle distribution for a case with superimposed excitation no matter whether it is in a fully filled state or in a partially filled state. Therefore, it can be predicted that the speed of sinusoidal pulsation would be beneficial for enhancing polymer processing.

The above conclusions, however, are drawn under the condition of specific vibration amplitude and frequency, so they have certain limitations. In follow-up research, more time would be spent on computing various cases of different speed excitation parameters for the fully filled state and the partially filled state. And the effect of different excitation speeds on the flow mixing is going to be analyzed to obtain the optimal operation speed in the near future. Secondly, various indicators to characterize the process of polymer mixing would be derived and perfected, such as the shearing rate, the stretching rate, the separation scale, the mixing index, and so on. Thirdly, more efforts would be made to develop a merging and splitting algorithm suitable for complex models like the twin screw component, then some real factors could be taken into consideration such as the temperature, the interfacial tension and surface tension, the shear heating, the viscoelastic properties, and the viscous dissipation in the polymer processing. Last but not least, the current work should be verified by performing further experiments. The first step is to build a simplified platform such as a 2D device, and then a real 3D platform is going to be built if the 2D simplified platform is successful.

## Figures and Tables

**Table 1:** Parameter setting of simulation models.

Item	Mark	Twin Screw	Barrel
Root diameter (mm)	*D_R_*	40	/
Crest diameter (mm)	*D_C_*	60	/
Root angle (degree)	*α*	22.88	/
Crest angle (degree)	*β*	22.88	/
Center line (mm)	*C_L_*	51	/
Lead/length (mm)	*L*	60	60
Gap between screws (mm)	*G*_1_	1	/
Gap between screw and barrel (mm)	*G*_2_	1	
Number of threads	*N_T_*	2	/
Inner diameter (mm)	*D*	/	62

**Table 2:** Initial simulation settings for fully filled and half-filled states.

Case No.	State	Excitation	Viscosity	*dp*	*dt*	$\rho_{0}$	*ω*	*f*	*A*	*c*	Number of Particles		
			Pa.s	mm	s	kg/m^3^	°/s	Hz	°	m/s	Total	Fluid	Boundary
1	fully filled	false	1	0.25	3.75 × 10^−6^	1000	360	0	0	20	9,990,642	8,824,766	1,165,876
2		true					360 + 6sin(10πt)	5	6				
3	half filled	false					360	0	0		5,563,596	4,397,720	
4		true					360 + 6sin(10πt)	5	6				

**Table 3:** Statistical results of max velocities and pressures for fully filled and half-filled states.

Case No.	State	Max Velocity	Delta	Max Pressure	Delta
		m/s	%	Pa	%
1	fully filled	0.455	30.1%	7842	19.8%
2		0.592		9392	
3	half filled	0.800	−10.4%	5452	23.5%
4		0.717		6731	

## Data Availability

The data presented in this study are available on request from the corresponding author. The data are not publicly available due to privacy reasons.

## Appendix A. Deduction of Equation (1)

Given any timestep *dt*, let $∆\theta_{b}$ and $∆\theta_{e}$ be the contributions of M1 and M2, respectively. Thus, we have $∆\theta_{b}=6N_{0}dt$, and

$$\begin{array}{cc} ∆\theta_{e} & =A\sin\left[2\pi f\left(t+∆t\right)\right]-A\sin\left(2\pi ft\right)\\ & =A{sin}^{\prime }\left(2\pi ft\right)dt\\ & =2\pi fAdtcos(2\pi ft) \end{array}$$

Since $cos\left(2\pi ft\right)\leq 1$, $∆\theta_{e}\leq 2\pi fAdt$.

It is common in polymer processing that $∆\theta_{e}$ cannot be greater than $∆\theta_{b}$; otherwise, the polymer particles may flow back into the screw channel. Then, we have $2\pi fAdt\leq 6N_{0}dt$, that is,

$$Af\leq 3N_{0}/\pi$$
